# Preliminary Outcome of Transcatheter Aortic Valve Implantation at Centers Without On-Site Cardiac Surgery

**DOI:** 10.3390/jcdd13060226

**Published:** 2026-05-27

**Authors:** Gianni Dall’Ara, Miriam Compagnone, Simone Grotti, Andrea Santarelli, Marco Balducelli, Caterina Cavazza, Carlo Savini, Carolina Moretti, Filippo Ottani, Andrea Rubboli, Marcello Galvani, Carmine Pizzi, Fabio Felice Tarantino

**Affiliations:** 1 Structural Cardiology Unit Forlì-Cesena, Morgagni-Pierantoni Hospital, 47121 Forlì, Italy; 2 Department of Medical and Surgical Sciences (DIMEC), University of Bologna, 40126 Bologna, Italy; 3 Cardiology Unit, Bufalini Hospital, 47521 Cesena, Italy; 4 Cardiology Unit, Santa Maria delle Croci Hospital, 48121 Ravenna, Italy; 5 Cardiology Unit, Infermi Hospital, 47923 Rimini, Italy; 6 Cardiovascular Department, Maria Cecilia Hospital, GVM Care & Research, 48033 Cotignola, Italy; 7 Cardiovascular Research Unit, Fondazione Cardiologica Sacco, 47121 Forlì, Italy; 8 Cardiology Unit, Morgagni-Pierantoni Hospital, 47121 Forlì, Italy

**Keywords:** transcatheter aortic valve implantation, transcatheter aortic valve replacement, cardiac surgery, complications, heart team

## Abstract

In many countries, patients’ access to transcatheter aortic valve implantation (TAVI) is limited by reimbursement issues or delayed admission to heart valve centers, thus increasing the risk of adverse events in wait-listed patients. The TAVI AT HOME (TAH) is a single-arm, multicenter study aiming to evaluate the safety and efficacy of transfemoral TAVI performed at centers without on-site cardiac surgery by expert operators. The primary endpoint is 30-day all-cause mortality. This study focuses on the run-in phase of the registry, which was required by the ethics committee to perform an interim safety analysis. The outcome of 20 TAH patients enrolled at three Italian centers from May 2023 to May 2024 was compared to 41 TAVI cases included in the permanent local registry, matching the TAH inclusion/exclusion criteria. The two groups had similar baseline characteristics. Significantly more patients in the TAH group were deemed at prohibitive risk (85.0% vs. 56.1%; *p* = 0.026) but had similar surgical risk scores. A self-expanding device was used in most cases (60.7%). Technical success did not differ between groups (95.0% in the TAH vs. 85.4%, *p* = 0.409). No deaths at 30 days (primary endpoint) were observed. The 1-year survival rate did not differ between groups. After interim data analysis, the ethics committee authorized the completion of the TAH enrollment and extension to other centers. The TAH approach might represent an alternative model to allow timely access to TAVI without compromising safety and effectiveness.

## 1. Introduction

Aortic stenosis is the most common heart valve disease requiring medical attention for replacement in high-income countries [1]. It affects approximately 5% of adults over the age of 65 years [2], and its clinical course is invariably progressive. If untreated, survival declines dramatically, with mortality rates approaching 50% within two years from symptom onset, and 97% at five years [3]. Both surgical aortic valve replacement (SAVR) and transcatheter aortic valve implantation (TAVI) have been shown to relieve symptoms, improve survival, and currently have a Class I recommendation for patients with symptomatic aortic stenosis [4,5,6]. Over the past decade, indications for TAVI have progressively expanded, leading to a substantial increase in procedural volume worldwide [7]. Healthcare systems must adapt to the growing demand for TAVI in order to prevent prolonged waiting time, which is associated with increased mortality [8,9,10]. Delay in TAVI may also result in worsening functional status [11,12] and is associated with a higher risk of hemodynamic deterioration requiring urgent intervention [13]. Currently, when a patient with a Heart Team indication for TAVI is evaluated at a center without on-site cardiac surgery, two main pathways are generally available. In the first, the patient is referred to the heart valve center for the procedure. In the second, TAVI is performed “in service” at a center with cardiac surgery by expert operators from the referring (spoke) hospital, who continue to manage the patient throughout the entire treatment course. In both scenarios, the number of procedures remains limited by the capacity of the heart valve center, leading to an increased waiting list. An alternative strategy is to perform TAVI directly at hospitals without on-site cardiac surgery, a condition that represents a paradigm shift in the healthcare system organization in many countries. However, the rapidly evolving TAVI technology and increasing operator expertise have significantly reduced peri-procedural complications, including the need for emergent cardiac surgery, with current rates below 0.5% [7,14,15,16,17,18,19,20,21]. Moreover, only a minority of major complications are ultimately managed surgically, and the outcome in these patients remains poor even at centers with on-site cardiac surgery capability.

The aim of the “Transfemoral Transcatheter Aortic Valve Implantation AT Hospital without on-site cardiac surgery: early clinical OutcoME in patients with prohibitive or high surgical risk” (TAVI AT HOME-TAH) study is to investigate the feasibility and safety of TAVI in centers without on-site cardiac surgery, in a carefully selected patient population defined by strict inclusion and exclusion criteria.

## 2. Methods

This is a single-arm, interventional, multicenter, non-profit study involving patients with symptomatic severe aortic stenosis who are candidates for transfemoral TAVI to be performed in centers without on-site cardiac surgery. The study was approved by the Ethics Committee of the Local Health Agency of Romagna (C.E.-ROM) and registered on ClinicalTrials.gov (NCT05886517) [22]. The indication for TAVI, as well as eligibility for study participation, is determined by the Heart Team. Patients with a high or prohibitive surgical risk are identified through calculation of the EuroSCORE II and STS score, combined with an overall clinical assessment performed by the cardiac surgeon. In accordance with current international recommendations, the Heart Team also considers the patient’s global clinical condition and comorbidities when evaluating candidacy for TAVI, excluding patients with an estimated life expectancy of less than 12 months. Study-specific exclusion criteria include contraindications to transfemoral access, bicuspid aortic valve, valve-in-valve TAVI, and instrumental characteristics, evaluated by CT angiography, associated with an increased risk of major complications. A dedicated multidisciplinary consultation involving the cardiac surgeon is scheduled to inform patients about the indication for TAVI, discuss the opportunity to participate in the study, and obtain written informed consent [23,24].

Patients who are excluded from scientific study or decline participation will follow the standard organizational pathway and undergo TAVI in a center with on-site cardiac surgery. Participating centers must provide a regular 24 h interventional cardiology service [25], with an overall procedural volume of at least >60 TAVI/year performed in heart valve centers. A vascular surgery unit and a level III intensive care unit must be available on site. At least one expert interventional cardiologist should be present, with experience as a first operator in ≥30 TAVI procedures per year for a minimum of 3 years. Additionally, they should have consistent experience in coronary revascularization procedures (>75 percutaneous coronary interventions/year), management of large-bore vascular access and peripheral complications [25]. Regular and well-structured Heart Team meetings must be ensured. The study is divided into two phases. The initial pilot phase includes enrollment of 20 patients (10% of the total expected sample size) and is specifically designed to evaluate procedural safety. Upon completion of this phase, an interim analysis is conducted by an independent Data Safety Monitoring Board. The predefined safety threshold is a maximum of four adverse primary outcome events, based on complication rates reported in previously published studies [22].

## 3. Data Analysis and Outcome Endpoints

All study variables are described using descriptive statistics: categorical variables are presented as absolute frequencies and percentages, whereas continuous variables are reported as mean and standard deviation or median with interquartile range, according to data distribution. We compared the outcome of patients included in the first phase of the TAVI AT HOME registry to those included in the permanent local registry, matching the TAH inclusion/exclusion criteria, who had undergone prosthesis implantation by the same team of operators at a heart valve center. Between-group comparisons were done using the Student-T or Kruskal–Wallis test for continuous variables, the chi-square or Fisher’s exact test for categorical ones. One-year overall survival was studied by Kaplan–Meier analysis. The significance threshold used for all tests is <0.05. All analyses were performed with SPSS version 20.0 (SPSS Inc., Chicago, IL, USA).

The primary endpoint is 30-day all-cause mortality. Secondary endpoints include the assessment of technical success and the evaluation of every single complication at 30 days from the procedure [24]. Technical success is defined according to VARC-3 consensus [24]:

- freedom from mortality;
- successful access, delivery of the device, and retrieval of the delivery system;
- correct positioning of a single prosthetic heart valve into the proper anatomical location;
- freedom from surgery or intervention related to the device (excluding pacemaker implantation) or to a major vascular or access-related, or cardiac structural complication.

## 4. Results

This analysis focused on the outcome of 20 patients enrolled at three Italian TAH centers from May 2023 to May 2024, compared to 41 matched patients included in the institutional registry who underwent TAVI performed by the same operator team at the reference center with on-site cardiac surgery from January 2021 to May 2023.

The median age of the overall population was 84.6 (81.1–89.1) years. Baseline characteristics were comparable between the two groups (Table 1). The two independent inclusion criteria, namely a high-risk score obtained by online calculators and a prohibitive global estimated risk given by the cardiac surgeon, overlapped in 19 cases. Notably, a significantly higher proportion of patients in the TAH group were classified as having prohibitive surgical risk compared with the control group (85.0% vs. 56.1%; *p* = 0.026). A self-expanding TAVI device was used in most cases (60.7%). Technical success rate was similar between groups (95.0% in the TAH group vs. 85.4% in the control group; *p* = 0.409). In the TAH cohort, one case (5.0%) of upward prosthesis migration occurred and was successfully managed with valve-in-valve implantation. Among patients in the comparison group, unsuccessful procedures (14.6%) were related to five vascular complications and one periprocedural myocardial infarction. The rate of permanent pacemaker implantation did not differ significantly between groups (25.0% vs. 26.8%; *p* = 0.879). No cases required conversion to open cardiac surgery.

Importantly, no deaths occurred within 30 days in either group, defining the study’s primary endpoint.

**Figure 1:**
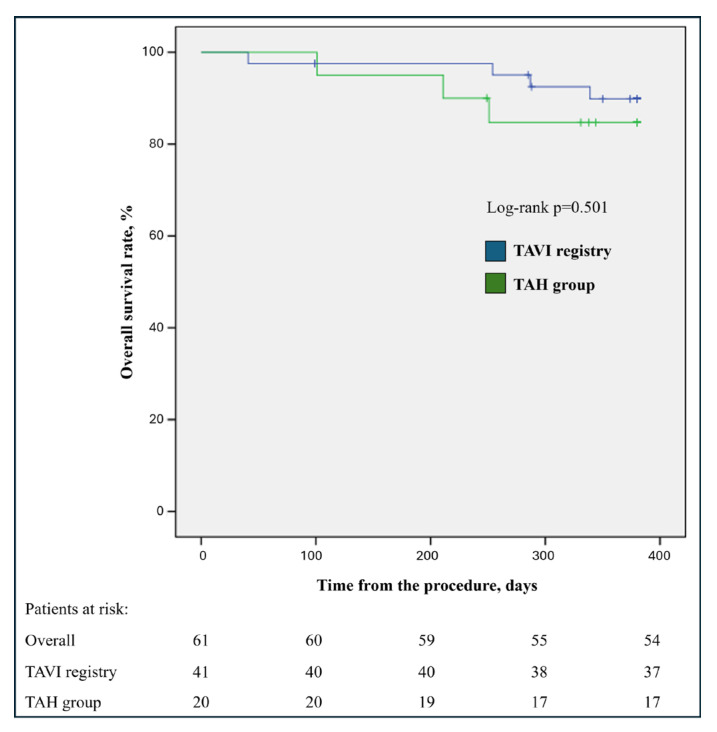
Analysis of 1-year overall survival.

The outcome analysis of patients enrolled during the TAH run-in phase demonstrated the absence of early mortality, with results comparable to those observed in a similar population undergoing TAVI at a heart valve center by the same experienced operators. Based on these findings, the Ethics Committee approved continuation of patient enrollment and expansion of the study to additional centers [22]. Furthermore, the one-year overall survival analysis showed no significant differences between groups (Figure 1).

## 5. Discussion

The aim of this study was to evaluate the safety and efficacy of transfemoral TAVI performed at centers without on-site cardiac surgery by expert operators with regular activity at a heart valve center. The main results of this run-in phase can be summarized as follows:

1. in the run-in TAH cohort, patients at high or prohibitive surgical risk undergoing TAVI in carefully selected centers without on-site cardiac surgery experienced no deaths within 30 days.
2. no significant differences were observed in terms of procedural technical success or complication rates compared with a matched population treated at a center with on-site cardiac surgery.
3. no severe procedural complications requiring conversion to open cardiac surgery occurred during the study period.

In many countries, the current volume of TAVI procedures remains below the estimated age-related clinical demand. Equitable access to treatment is primarily limited by reimbursement constraints, although delay in admission to heart valve centers also contributes significantly [26]. As a consequence, waiting time for TAVI may exceed three months, thereby increasing the risk of adverse events, including death [27]. Several strategies have been proposed to address this issue, depending on local logistics and the healthcare organization. These include: expanding the capacity of established heart valve centers, implementing “in-service” procedures for patients transferred from peripheral hospitals, and performing TAVI in centers without on-site cardiac surgery [28]. Current international guidelines recommend that TAVI be performed in heart valve centers, provided with on-site cardiac surgery. However, the need for bailout surgery after TAVI is uncommon, occurring in approximately 0.5% of cases, and it is associated with very high mortality rates [29].

During the early TAVI experiences in Germany, between 2010 and 2015, 639 procedures (4% of 15,964 TAVI) were performed in centers without on-site cardiac surgery, with a cardiac surgeon on standby [30]. In 2014, Eggebrecht et al. compared 1254 patients treated in 27 cardiac surgery hospitals with 178 patients (12%) treated at eight centers without on-site cardiac surgery. The authors found no significant differences in major post-procedural complication rates between the two groups. Specifically, 30-day all-cause mortality was similar between hospitals with cardiac surgery and those without (8.3% vs. 6.2%, respectively) [31]. Similarly, the AQUA registry, the largest study available on this topic, including 17,919 TAVI, of which 1332 were in non-surgical centers, demonstrated similar in-hospital mortality between patients treated in centers with or without on-site cardiac surgery (4.2% vs. 3.8%, respectively, *p* = 0.396). Rates of major complications and emergent cardiac surgery were also not statistically different between the two groups [32]. In a propensity-matched study published in 2018, 290 patients treated in non-surgical centers were compared with an equal number treated in hospitals with on-site cardiac surgery. No significant differences were observed in either short-term (30-day) or long-term (3-year) survival between groups [33]. Furthermore, a meta-analysis included 21,173 patients, of whom 1800 (8.5%) underwent TAVI in centers without on-site cardiac surgery, found comparable short-term mortality between the two settings (OR 1.05; 95% CI 0.64–1.71) [34]. Nevertheless, the debate regarding the optimal organizational model for TAVI care remains ongoing, and contemporary evidence is still limited [34].

**Figure 2:**
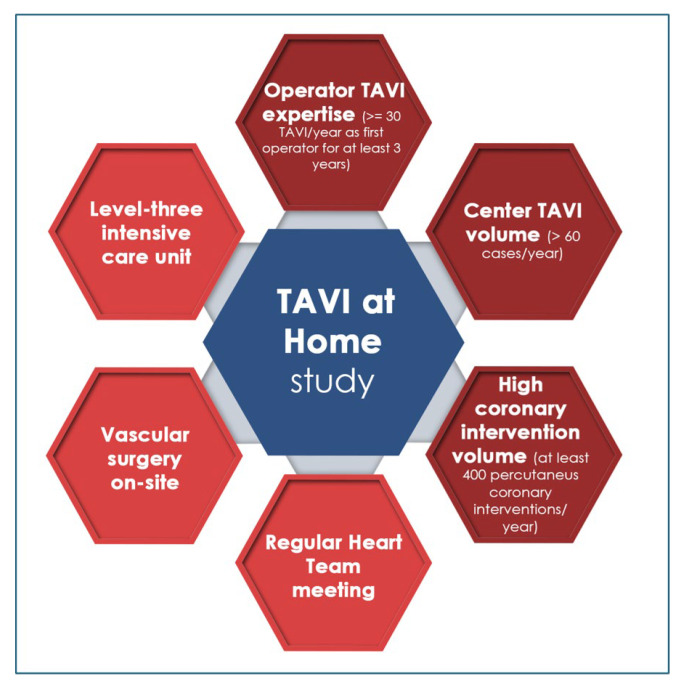
TAVI AT HOME study operators and centers’ requirements.

TAVI AT HOME is a single-arm, multicenter, investigator-initiated study designed to evaluate the safety and efficacy of transfemoral TAVI performed in centers without on-site cardiac surgery by experienced operators with established activity at a dedicated heart valve center (Figure 1) in the contemporary era [22]. Eligible patients included those considered at high or prohibitive surgical risk, as determined during Heart Team discussions. This likely explains the higher proportion of prohibitive-risk patients observed in the TAH group, since this classification may depend on clinical features not fully captured by conventional surgical risk calculators (Table 1). Importantly, all other baseline characteristics, including comprehensive surgical risk scores, were similar between groups, supporting the comparability of the two study populations. The observed complication rates were consistent with those reported in previous studies involving elderly and frail patients at high or prohibitive surgical risk [35]. Importantly, TAH hospitals are integrated within a hub-and-spoke network, allowing rapid transfer to a reference cardiac surgery center in the event of major complications. Such organizational measures are essential to mitigate rare but potentially catastrophic complications and to maintain procedural safety. Notably, no cases requiring conversion to open cardiac surgery occurred in the present analysis. This study includes a relatively small and highly selected patient population and therefore shares the limitations inherent to non-randomized observational studies. Consequently, these findings should be confirmed in larger-scale studies with adequate statistical power and randomized design. On the other side, the rationale of the TAH model is strengthened by recent evidence showing a progressive decline in the need for emergency cardiac surgery after TAVI, currently estimated at approximately 0.25% [29]. Some life-threatening complications, such as valve annulus rupture, are sometimes excluded from bailout surgery, while mortality associated with emergent surgical conversion remains extremely high, approaching 50% [21]. Importantly, many procedural complications can be managed percutaneously or by means of vascular surgery. The aim of the TAH study is to identify patients and logistic characteristics to establish an efficient and safe TAVI network where procedures can be performed at hospitals without on-site cardiac surgery. Patients at high and prohibitive risk might be eligible at the beginning of the program, at hospitals fulfilling the characteristics of TAH centers, and located within 30–60 min of a reference cardiac surgery center (Figure 2).

From a health system perspective, this organizational model may help overcome structural barriers limiting timely access to TAVI in regions with insufficient heart valve center capacity. Considering that mortality among patients waiting longer than three months for TAVI has been estimated at approximately 2.5%, the TAH approach may represent a scalable strategy to reduce preventable deaths through decentralization of care without compromising procedural safety.

## 6. Conclusions

The TAH model might represent an option to allow timely access to TAVI while ensuring procedural efficacy and safety. Comprehensive and robust data are desirable on this topic.

## Figures and Tables

**Table 1:** Baseline characteristics, procedural data, and outcomes.

	Overall *n* = 61	TAVI at HOME *n* = 20	TAVI Registry *n* = 41	*p*
Age, years	84.6 (81.1–89.1)	86.9 (85.0–89.2)	86.1 (79.0–89.1)	0.181
Male gender	33 (54.1)	13 (65.0)	20 (48.8)	0.233
Hypertension	51 (83.6)	16 (80.0)	35 (85.4)	0.716
Diabetes	17 (27.9)	5 (25.0)	12 (29.3)	0.727
Body mass index, kg/m^2^	25.4 ± 5.2	24.5 ± 4.6	25.7 ± 5.40	0.897
Atrial fibrillation	13 (21.3)	5 (25.0)	8 (19.5)	0.741
Previous PCI	19 (31.1)	6 (30.0)	13 (31.7)	0.892
Previous CABG	11 (18.0)	5 (25.0)	6 (14.6)	0.479
Previous stroke	7 (11.5)	3 (15.0)	4 (9.8)	0.674
Dialysis	4 (6.6)	1 (5.0)	3 (7.3)	1.000
Severe COPD	3 (4.9)	2 (10.0)	1 (2.4)	0.248
Active cancer	9 (14.8)	4 (20.0)	5 (12.2)	0.458
Hemoglobin, g/dL	11.7 ± 2.1	11.9 ± 1.4	11.5 ± 2.3	0.506
Creatinine, mg/dL	1.0 (1.0–2.0)	1.0 (1.0–2.0)	1.00 (1.0–2.0)	0.747
Echo data				
LVEF, %	56.0 (45.0–60.0)	55.5 (37.5–60.0)	56.0 (45.0–60.0)	0.975
aortic valve area, cm^2^	0.82 ± 0.40	0.74 ± 0.21	0.74 ± 0.16	0.916
mean gradient, mmHg	43.0 (39.5–50.5)	40.5 (38.3–46.8)	44.0 (40.0–51.5)	0.410
PAP, mmHg	30.0 (30.0–40.0)	30.0 (26.3–40.0)	30.0 (30.0–39.0)	0.872
High surgical risk *	40 (65.6)	13 (65.0)	27 (65.9%)	0.947
STS score	7.0 (5.0–9.0)	8.5 (5.0–12.8)	7.0 (3.5–8.0)	0.087
Euroscore II	7.0 (5.0–9.0)	7.0 (4.0–10.0)	7.0 (4.0–8.0)	0.737
Prohibitive risk *	40 (65.6)	17 (85.0)	23 (56.1)	0.026
porcelain aorta		4 (20.0)	4 (9.8)	
frailty		7 (35.0)	6 (14.6)	
CABG/hostile thorax		5 (25.0)	7 (17.1)	
comorbidities		1 (5.0)	6 (14.6)	
Urgent TAVI	12 (19.7)	5 (25.0)	7 (17.1)	0.505
Prosthesis type				0.628
balloon-expandable	24 (39.3)	7 (35.0)	17 (41.5)	
self-expanding	37 (60.7)	13 (65)	24 (58.5)	
Technical success	54 (88.5)	19 (95.0)	35 (85.4)	0.409
Conversion to open surgery	0 (0.0)	0 (0.0)	0 (0.0)	n.a.
Vascular complications	12 (19.7)	2 (10)	10 (24.4)	0.305
minor	7 (11.5)	1 (5.0)	6 (14.6)	0.409
major	5 (8.2)	1 (5.0)	4 (9.8)	1.000
Acute neurological events	1 (1.6)	1 (5.0)	0 (0.0)	0.328
minor stroke	1 (1.6)	1 (5.0)	0 (0.0)	0.328
stroke	0 (0.0)	0 (0.0)	0 (0.0)	n.a.
Myocardial infarction	2 (3.3)	1 (5.0)	1 (2.4)	1.000
Bleeding types (BARC)	17 (27.9)	6 (30.0)	11 (26.8)	0.795
type 1–2	15 (24.6)	5 (25.0)	10 (24.4)	1.000
type 3–4	2 (3.3)	1 (5.0)	1 (2.4)	1.000
Permanent PM implantation	16 (26.2)	5 (25.0)	11 (26.8)	0.879
Hospital stay, days	7.0 (4.0–11.0)	9.0 (6.5–11.3)	25.0 (22.2–27.8)	0.099
Deaths at follow-up				
30-day	0 (0.0)	0 (0.0)	0 (0.0)	n.a.
1-year	6 (9.8)	3 (15.0)	3 (7.3)	0.384

Continuous variables are expressed as mean ± standard deviation or median (interquartile range); categorical variables as number (%). BARC: Bleeding Academic Research Consortium; CABG: coronary artery bypass graft; COPD: chronic obstructive pulmonary disease; LVEF: left ventricular ejection fraction; PAP: pulmonary artery pressure; PCI: percutaneous coronary intervention; PM: permanent pacemaker; STS: Society of Thoracic Surgeons; TAVI: transcatheter aortic valve implantation. * More options are possible as inclusion criteria.

## Data Availability

Anonymized data will be made available by the corresponding author upon reasonable request.
